# Cobalt-containing borate bioactive glass fibers for treatment of diabetic wound

**DOI:** 10.1007/s10856-023-06741-3

**Published:** 2023-08-02

**Authors:** Minhui Zhang, Aihua Yao, Fanrong Ai, Jian Lin, Qingge Fu, Deping Wang

**Affiliations:** 1 School of Materials Science and Engineering, Tongji University, 200092 Shanghai, China; 2 Key Laboratory of Advanced Civil Engineering Materials, Ministry of Education, 200092 Shanghai, China; 3 School of Mechatronics Engineering, Nanchang University, 330031 Nanchang, China; 4 Department of Orthopedic trauma, Changhai Hospital, Second Military Medical University, 200433 Shanghai, China

## Abstract

Impaired angiogenesis is one of the predominant reasons for non-healing diabetic wounds. Cobalt is well known for its capacity to induce angiogenesis by stabilizing hypoxia-inducible factor-1α (HIF-1α) and subsequently inducing the production of vascular endothelial growth factor (VEGF). In this study, Co-containing borate bioactive glasses and their derived fibers were fabricated by partially replacing CaO in 1393B3 borate glass with CoO. Fourier transform infrared (FTIR) spectroscopy and nuclear magnetic resonance (NMR) analyses were performed to characterize the effect of Co incorporation on the glass structure, and the results showed that the substitution promoted the transformation of [BO_3_] into [BO_4_] units, which endow the glass with higher chemical durability and lower reaction rate with the simulated body fluid (SBF), thereby achieving sustained and controlled Co^2+^ ion release. In vitro biological assays were performed to assess the angiogenic potential of the Co-containing borate glass fibers. It was found that the released Co^2+^ ion significantly enhanced the proliferation, migration and tube formation of the Human Umbilical Vein Endothelial Cells (HUVECs) by upregulating the expression of angiogenesis-related proteins such as HIF-1α and VEGF. Finally. In vivo results demonstrated that the Co-containing fibers accelerated full-thickness skin wound healing in streptozotocin (STZ)-induced diabetic rat model by promoting angiogenesis and re-epithelialization.

**Graphical Abstract:**

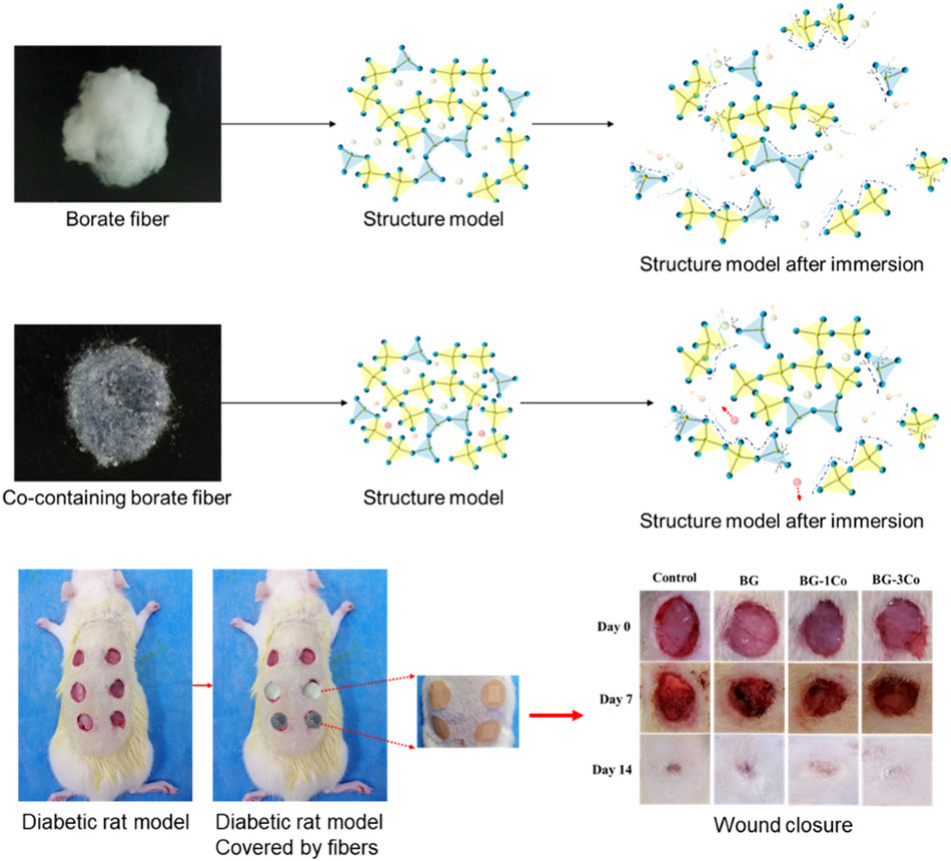

## Introduction

With a rapid aging of global population and the improvement of people’s living standards, the prevalence of diabetes has increased at an alarming rate in the recent decades, causing a heavy global burden on the public health and economic development [1]. It has been established that abnormally high blood glucose levels can lead to various complications in diabetic patients. Diabetic foot ulcer (DFU) is the most severe chronic complication of poorly controlled diabetes [2]. Impaired angiogenesis in diabetic wound can lead to chronic hypoxia and inadequate micronutrient delivery, which can amplify inflammation and impede the process of wound healing [3]. Hence, novel strategies to effectively promote local angiogenesis have been designed to accelerate the healing process of diabetic wounds. Local administration of angiogenesis-related factors, such as platelet derived growth factor (PDGF) and vascular endothelial growth factor (VEGF) have been found to be effective in accelerating neovascularization and stimulating wound healing in experimental diabetic models [4]. However, due to the short half-lives or low protein stability of vascular growth factors, they have exhibited limited efficacy and potential risk associated with their overdose have hampered their clinical applications. Alternatively, some inorganic ionic species, such as Cu^2+^ and Co^2+^, have been reported to stimulate angiogenesis in a safer and more cost-effective way, and thus are considered as a possible alternative to growth factors and genetic approaches [5].

A number of studies have shown that impaired hypoxia-inducible factor-1 (HIF-1) activity in diabetic wounds can lead to insufficient production of VEGF, and thus decreased blood vessel density and poor blood circulation. HIF-1 is heterodimeric complex consisting of HIF-1α and HIF-1β, among which the HIF-1β is found in all cells, whereas HIF-1α is oxygen-regulated subunit [6]. High glucose levels can destabilize HIF-1α, leading to inadequate wound healing in diabetic patients [7]. Therefore, stabilizing HIF-1α is essential for improving wound healing process in diabetic environment. In addition, it has been confirmed that the activation of HIF activity produces a more physiological and “balanced” angiogenic response than the use of a single growth factor for therapeutic angiogenesis [1]. Cobalt is well known for its ability to activate HIF-1 pathway by preventing hydroxylation of HIF-1α [8]. For instance, E. Bosch-Rué et al. [9] reported that in vitro angiogenesis could be substantially promoted when treating Human Umbilical Vein Endothelial cells (HUVECs) with non-toxic concentrations (≤50 μM) of Co^2+^ (supplied by CoCl_2_ solution), due to the upregulation of HIF-1α, VEGF and platelet endothelial cell adhesion molecule-1 (PECAM-1). Also, there existed a significant correlation between HIF-1α and VEGF expression. However, like other trace element, Co is also reported to be toxic when used at high dosage, and thus their controlled release from suitable carrier system is critical [10]. Owing to their amorphous structure, bioactive glasses allow a large flexibility in composition, which makes them possible to incorporate varying concentrations of therapeutically active ions. Upon degradation of the bioactive glasses in the biological fluids, these ions are released at varying rates depending on the glass compositions and heat treatment conditions. For example, Co^2+^ ions were released from the Co-containing melt-derived 13-93 bioactive glass scaffolds at rates (after the initial burst release) of between 0.3 ppm/day and 0.1 ppm/day depending on CoO content in the glass network [5, 10]. Cell biology studies showed that inclusion of 1 wt% CoO was biocompatible with osteoblast-like cell and endothelial cells, and also the released Co^2+^ significantly promoted the formation of tube-like structures. However, addition of 5 wt% CoO (released ~12 ppm Co^2+^) was cytotoxic to both cell types [5].

Compared to silicate bioactive glasses, some borate-based bioactive glasses degrade more quickly and completely due to their lower chemical durability. Also, despite fast conversion to CaP, borate bioactive glasses convert to hydroxyapatite (HA) more slowly than silicate glasses [11], which makes them more suitable for wound healing application, as the formation of HA layers on the surface of bioactive glass is undesirable in soft tissue repair [12–14]. Besides, the positive effects of boron on wound healing have been validated. For example, boron has been shown to promote angiogenesis, take part in the synthesis of extracellular matrix and stimulate secretion of collagen, proteoglycans and proteins, et al. [15]. Borate bioactive glasses 13-93B3 (54B_2_O_3_-22CaO-6Na_2_O-8K_2_O-8MgO-2P_2_O_5_, mol%) derived from silicate bioactive glass 13-93 (54SiO_2_-22CaO-6Na_2_O-8K_2_O-8MgO-2P_2_O_5_, mol%) has shown significant promise in accelerating wound closure in healthy rats. In 2016, cotton -like nanofibers composed of the 13-93B3 glasses were approved by the U.S. Food and Drug Administration (FDA) as a novel wound dressing for the treatment of acute and chronic wounds [16]. Recently, bioactive borate glass fibers have also shown their clinical potential in treatment of chronic diabetic wounds [17].

In addition to the biological effects of the therapeutic ions, their incorporation can significantly influence the network connectivity of original glass, and thus the associated properties, such as degradation behavior, ion release kinetics as well as apatite formation ability, will be affected. CoO is known to act in a concentration dependent manner in silicate glasses as both network former and network modifier [18, 19]. Therefore, partially replacement of modifier oxides (such as CaO) with CoO may result in a more connected SiO_2_ network, and consequently a slower degradation rate. However, when CoO is only used as a network modifier, the SiO_2_ network is depolymerized through the formation of non-bridging oxygen, thereby accelerating glass degradation and ion release.

The main purpose of the present study is to investigate the roles of Co^2+^ released from the 13-93B3 bioactive glass-derived fibers in accelerating diabetic wound healing. Due to cation field strength effect, Co^2+^ has important but complex effects on boron coordination, which will have significant effects on the physicochemical properties of the borate glass. Therefore, the influences of Co incorporation on the network structure and properties of the borate bioactive glass were firstly systematically discussed. Thereafter, in vitro biological assays were performed to assess the cytocompatibility of the Co-containing borate glass fibers and their angiogenic potential. Finally, their wound healing potential was confirmed in streptozotocin (STZ)-induced diabetic rat models.

## Materials and methods

### Preparation of Co-containing borate bioactive glass fibers

The Co-containing borate bioactive glasses with varying Co contents with the compositions of 6Na_2_O-8K_2_O-8MgO-(22-x)CaO-xCoO-54B_2_O_3_-2P_2_O_5_ (mol%) (x = 0, 1, 2, 3 and 5, defined as BG, BG-1Co, BG-2Co, BG-3Co and BG-5Co, respectively) were prepared by melting the requisite amounts of Na_2_CO_3_, K_2_CO_3_, (MgCO_3_)_4_·Mg(OH)_2_·5H_2_O, CaCO_3_, H_3_BO_3_, NH_4_H_2_PO_4_, CoCl_2_·6H_2_O and CuSO_4_·5H_2_O (analytical grade; Sinopharm Chemical Reagent Co., Ltd. China) in a platinum crucible for 1 h at 1150 °C.

The borate bioactive glass fibers were produced by blowing the melts using high-pressure (0.35 MPa) cold air when the melts were poured from a crucible at a rate of 0.10 L/s. Thereafter, the melts were left for cooling at the room temperature and the cotton-like glass fibers were collected.

### Characterization of borate bioactive glass fibers

The borate glasses and the products formed during in vitro immersion were investigated using Fourier transform infrared (FTIR) spectrometer (Impact 380 Nicolet, Thermo Fisher Scientific, USA). For that, the pellets were made by pressing the mixture of 1 mg sample powders and 100 mg potassium bromide (KBr). Spectra were then recorded between 4000 and 400 cm^−1^ with a resolution of 4 cm^−1^. ^11^B MAS-NMR was used to analyze the glass samples using a Varian 14.1 Tesla spectrometer (Varian Inc. Palo Alto, CA) equipped with a Varian/Chemagnetics T3 probe. The samples were packed in a 4 mm zirconia rotors spinning at 20 kHz.

### In vitro degradation and ion release behavior

0.1 g of glass fibers were immersed in 30 mL of simulated body fluid (SBF), SBF was configured according to the method described by Kokubo et al. [20]. At predetermined time intervals, the samples were removed from SBF and washed with deionized water. The fibers were powdered, and the phase composition of the conversion products was detected using X-ray diffractometer (XRD, Smartlab 9, Rigaku, Japan) in a 2θ range of 20–80° with Cu Kα radiation. The Co^2+^ released from the glass fibers were detected by inductively-coupled plasma atomic emission spectroscopy (ICP-AES; Optima 2100 DV; USA). Scanning electron microscopy equipped with Energy Dispersive Spectrometer (SEM-EDS; Model Quanta 200 FEG, FEI Co.) was used to observe the surface morphology of the fibers.

### Cell culture and ionic dissolution products of the glass fibers

Human umbilical vein endothelial cells (HUVECs) and human skin fibroblasts (HSF) were approved by the ethical committee of the Second Affiliated Hospital of Nanchang University. Both cells were cultured in α minimum essential medium (α-MEM, Gibco) supplemented with 10% (v/v) fetal bovine serum (FBS, Gibco), and maintained at 37 °C in a humidified atmosphere containing 5% CO_2_.

The ionic dissolution products of the BG and Co-containing BG glass fibers were prepared separately by soaking 0.5 g of fibers in serum-free Dulbecco’s modified Eagle’s medium (DMEM). After incubation at 37 °C for 24 h, the mixture was centrifuged and the supernatant was collected, filtered through a 0.2 μm syringe filter, and then diluted to different concentrations.

### In vitro biocompatibility and angiogenesis evaluation of the ionic dissolution products

#### In vitro cytotoxicity test

Cell Counting Kit-8 (CCK-8) assay was used to evaluate the cytotoxicity of the glass dissolution products on HUVECs and HSF following ISO993-5 standard. The ionic extracts with concentrations of 2000, 500 and 125 μg/mL were used as experimental groups and the culture media without glass extract was used as blank control. 100 μL of cell suspensions were seeded in 96-well culture plates at a density of 2 × 10^5^ cells/well. Subsequently 10 μL of extracts were added to each well. On day 1, 3 and 7, the culture media were removed and 100 μL of serum-free DMEM containing 10 μL of CCK-8 (Beyotime Biotechonology Ltd., China) reagent was added and incubated at 37 °C for another 2 h. After that, aliquots (100 μL) were taken from each well and transferred to another 96-well plate. The optical density (OD) was measured at a wavelength of 450 nm using a spectrophotometric microplate reader (Bio-Rad 680, USA).

Calcein-AM/PI double stain kit (Yeasen Biotechonology Ltd. China) was used to further evaluate the in vitro cytotoxicity. HUVECs were seeded in 24-well plates at an initial density of 5 × 10^3^ cells/well. The extracts were added to each well and incubated at 37 °C for 1 and 3 days. The cells were then incubated with a dying fluid consisting of 2 μM Calcein-AM and 4.5 μM PI for 20 min at 37 °C, and mounted on a glass coverslip. Live and dead cells were identified by green and red fluorescence, respectively, using a fluorescence microscope (Leica Microsystems, Mannheim, Germany).

#### Scratch-wound assay

HUEVCs were seeded in 6-well plates at a density of 4 × 10^5^ cells/well, and incubated in the medium supplemented with 10% FBS at 37 °C. When cell confluence reached ~90%, a 10 μL pipette tip was used to generate scratch zones by streaking the culture plate perpendicularly. The Cells were gently washed with PBS three times to remove the debris and smoothen the edges of the scratch. After that, the culture medium was replaced with the glass extracts and the cells were cultured in CO_2_ at 37 °C. The scratch zones were observed at different time points (12 and 24 h) to monitor the migration of cells. The results were analyzed using ImageJ software and the percentage of gap closure was calculated.

#### Transwell migration assay

Transwell inserts (8 μm pore-size filter membranes; Corning, NY, USA) were incubated with 100 μL of diluted Matrigel (Corning, NY, USA) for 1 h at 37 °C. HUEVCs were digested with trypsin, and 200 μL of serum-free DMEM containing 5 × 10^4^ cells was added to the upper chamber of Transwell. In the lower chamber of the Transwell, 500 μL CSGS culture medium with 15% serum was added. The glass extracts at a concentration of 0.125 mg/mL were added to the upper chamber. After 48 h of incubation, the chamber was taken out and the culture plate was washed with PBS three times. The migrated cells adhering to the bottom side of the Transwell membrane were fixed with 4% paraformaldehyde for 30 min, stained with crystal violet staining solution (Solarbio, Beijing) for 20 min. The migrated cells were observed under an inverted microscope (Leica Microsystems, Mannheim, Germany). Cells in five random fields were counted for each sample using ImageJ software.

#### Matrigel-based tube formation assay

To evaluate the angiogenic potential of the HUVECs incubated in different extracts, a matrigel tube formation assay was performed. After thawing on ice at 4 °C overnight, 50 μL Matrigel (Corning Inc. NY, USA) was added to a pre-cooled 96-well plate and incubated at 37 °C for 60 min to form a gel. HUEVCs were seeded in each Matrigel-coated well at a density of 2 × 10^5^ cells/well and incubated in conditioned media at 37 °C. After gelation, 50 μL of cell suspension was added to each coated well, and then 50 μL of various concentrations of glass extracts were added. After 6 h of incubation, five fields were randomly selected to observe network structural complexity, and the number of tubes was analyzed using ImageJ software.

#### Western blotting analysis

Western blotting analysis was performed to detect the protein expression levels of HIF-1α and VEGF. HUVEC suspensions were added into the glass extracts with a final concentration of 2 mg/mL, and incubated for 24 h in CO_2_ at 37 °C. After that, the cells were harvested and lysed on ice with RIPA lysis buffer (Sigma-Aldrich) with protease inhibitors and phosphatase inhibitor cocktails (Roche, Germany). The cell lysates were centrifuged for 10 min at 15000 *rpm* at 4 °C, and the supernatant was collected. The total protein concentration was determined by the Bio-Rad protein assay. Equal amounts of proteins were then separated by 10% SDS-polyacrylamide gel electrophoresis, and the separated proteins were electrically transferred onto polyvinylidene difluoride (PVDF) membranes (Millipore, USA). After being blocked with 5% non-fat milk, the blots were incubated overnight with primary antibodies of anti-human HIF-1α (ab51608, 1:1000, Abcam) and VEGF (ab51745, 1:2000, Abcam), respectively, and GAPDH (ab9484, 1:1000, Abcam) as the loading control. The blots were then incubated at room temperature for 1 h with appropriate HRP conjugated secondary antibodies. The membrane was exposed to ECL substrate solution for 5 min, and the signals were visualized by a ChemiDoc-XRS imaging system (Biorad). Signal intensity was analyzed using Image J.

### In vivo evaluation of diabetic wound healing

#### Animal experiments

Twelve adult Sprague Dawley (SD) rats were used in this study. All animal experimental were conducted in accordance with the National Institutes of Health Guide for the Care and Use of Laboratory Animals and approved by the Biological Research Ethics Committee of the Second Affiliated Hospital of Nanchang University. Diabetes were induced in the rats with STZ injected intraperitoneally once at a dose of 50 mg/kg. The level of blood glucose was measured on day 3 and day 5, and the rats with fasting blood glucose levels higher than 16.7 mmol/L were selected as diabetic. The STZ-induced diabetic rats were anesthetized with chloral hydrate sodium via intraperitoneal injection, and dorsum were shaved. The wound area were marked and then sterilized with iodine prior to incision. The excisional wound model was established based on previous method [21]. Six circular full-thickness excisional wounds, 10 mm in diameter were made on the dorsum with an experienced surgeon, without damaging the fascia. Circular silicone sheets were attached over the wound, and 0.1 g of various compositions of glass fiber samples (BG, BG-1Co, BG-2Co and BG-3Co) were then separately placed on each skin defect, while the last one was used as a control and left empty. After the surgery, Band-Aids were placed over the wounds to cover and fix the fiber samples.

#### Wound closure measurement

On days 7 and 14 post-surgery, the Band-Aids on the wounds were removed, and each defect was photographed by a digital camera. The size of the wound was analyzed by ImagePro Plus 4.5 software. The rate of wound closure was defined as the ratio of the wound size to the initial wound size.

#### Histology analysis

The explanted tissue was fixed in 4% paraformaldehyde at 4 °C overnight, and then dehydrated in 30% sucrose solution. After embedded in OCT, the explants were cut into 5 μm thick sections. Subsequently the sections were stained with Hematoxylin & Eosin (H&E) to evaluate re-epithelialization.

### Statistical analysis

All statistical analyses were conducted through a one-way analysis of variance (ANOVA). The data were presented as the mean ± standard deviation. Differences among groups were analyzed and the differences were considered to be statistically significant if *p* < 0.05.

## Results

### Structure study of Co-containing borate bioactive glass

**Fig. 1:**
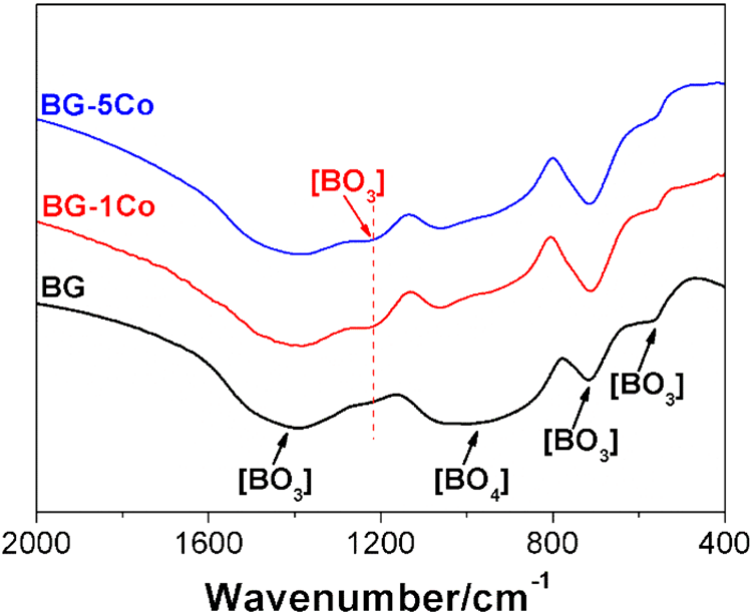
FTIR spectra of different compositions of borate bioactive glasses

Cobalt was incorporated into 1393B3 borate bioactive glass by replacing CaO with CoO up to 5 mol%, and the effect of Co incorporation on the structure of borate glass was investigated using FTIR and ^11^B MAS-NMR. Figure 1 shows FTIR spectra of the BG, BG-1Co and BG-5Co glasses. The broad bands in the range 1300–1600 cm^−1^ and 800–1150 cm^−1^ are attributed to B-O stretching modes of both trigonal [BO_3_] and tetrahedral [BO_4_] units in the borate structures, respectively. The bands at 712 cm^−1^ and 560 cm^−1^ correspond to the bending vibration of B-O-B in symmetric [BO_3_] triangles [22–25]. It is noted that the strength of the band at ~1220 cm^−1^ increases with increasing CoO content. It was reported that the band at around 1220 cm^−1^ was associated with the stretching vibration of the B-O bonds of [BO_3_] units involving mainly the linkage oxygen connecting different groups [26].

**Fig. 2:**
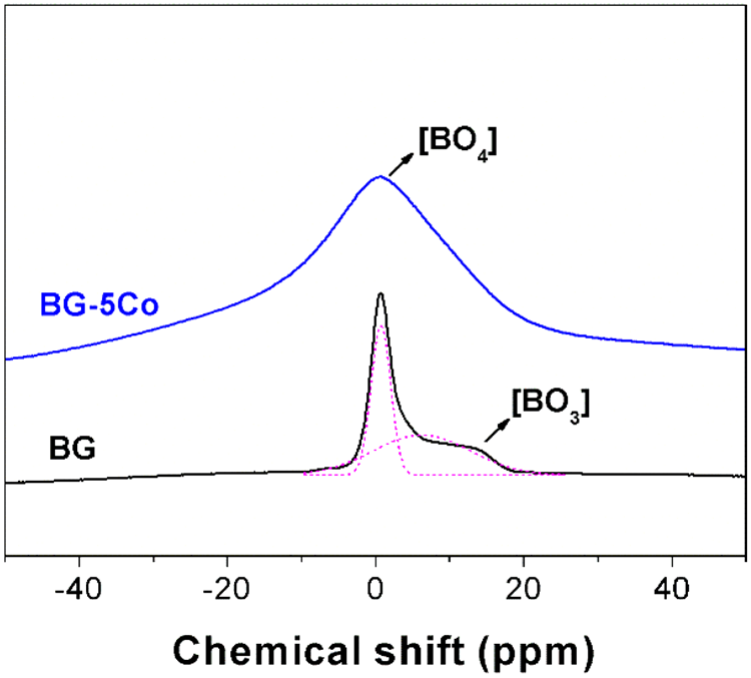
^11^B MAS-NMR spectra of different compositions of borate bioactive glasses

^11^B MAS-NMR spectra of both BG and BG-5Co glasses are presented in Fig. 2. The spectrum of Co-free BG glass reveals one narrow ^11^B resonance in the lower shift range and a broad counterpart in the high-ppm region, corresponding to higher/lower local symmetry of B in the tetrahedral [BO_4_] and planar [BO_3_] units, respectively [27]. However, the spectrum of BG-5Co glass only contains a broadened peak belonging to [BO_4_] units, and the peak corresponding to [BO_3_] units was not found. It should be explained that the broadening of the NMR signals is ascribed to the paramagnetism of Co^2+^ ions present in the glass structure [28–30].

### In vitro biological activity of Co-containing borate glass fiber

#### Degradation property

**Fig. 3:**
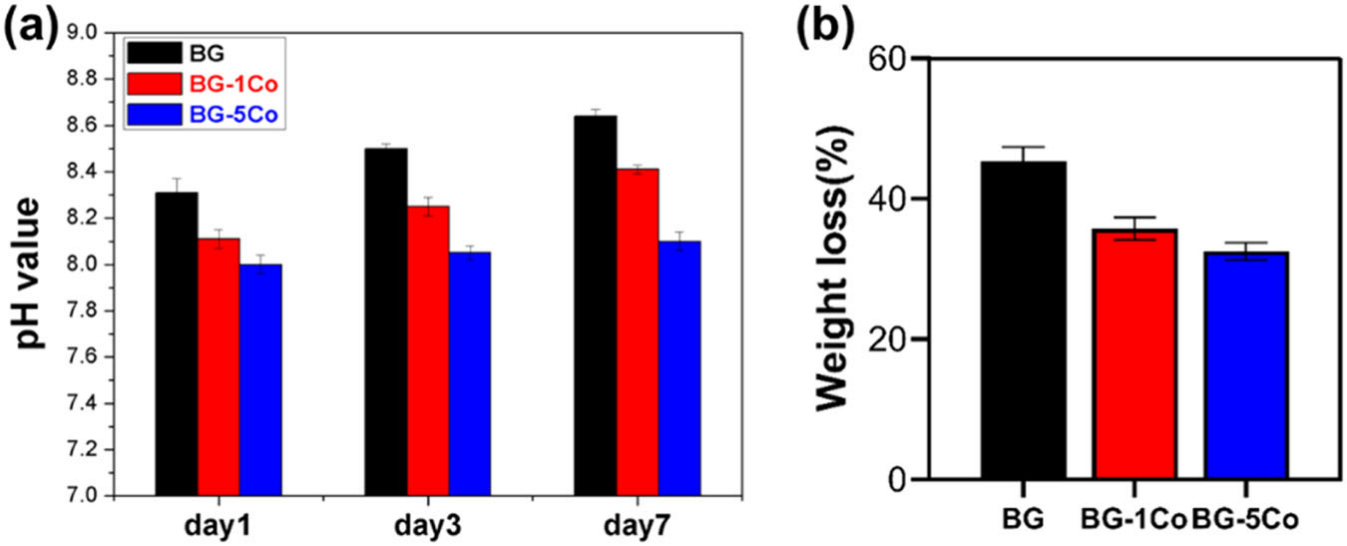
**a** pH variation of SBF and (**b**) weight loss during the degradation of different compositions of borate bioactive glass fibers

In vitro degradation behavior of the as-prepared borate glass fibers were evaluated by immersion in SBF at 37 °C, and the pH variation of SBF was monitored. As shown in Fig. 3a, for BG fibers, the pH of the SBF increases rapidly from 7.45 to 8.3 during the first day of immersion due to rapid exchange of alkali and alkaline-earth cations with H^+^ ions from the solution, and then the pH values increase continuously with immersion time, eventually reaching ~8.7 on day 7. However, the speed of pH rising rate decreases with increasing CoO content. Figure 3b shows the weight loss of BG, BG-1Co and BG-5Co fibers after immersion in SBF for 7 days. The mass loss of fibers decreases with the increasing amount of CoO, which is consistent with the pH results.

#### Ion release behavior

**Fig. 4:**
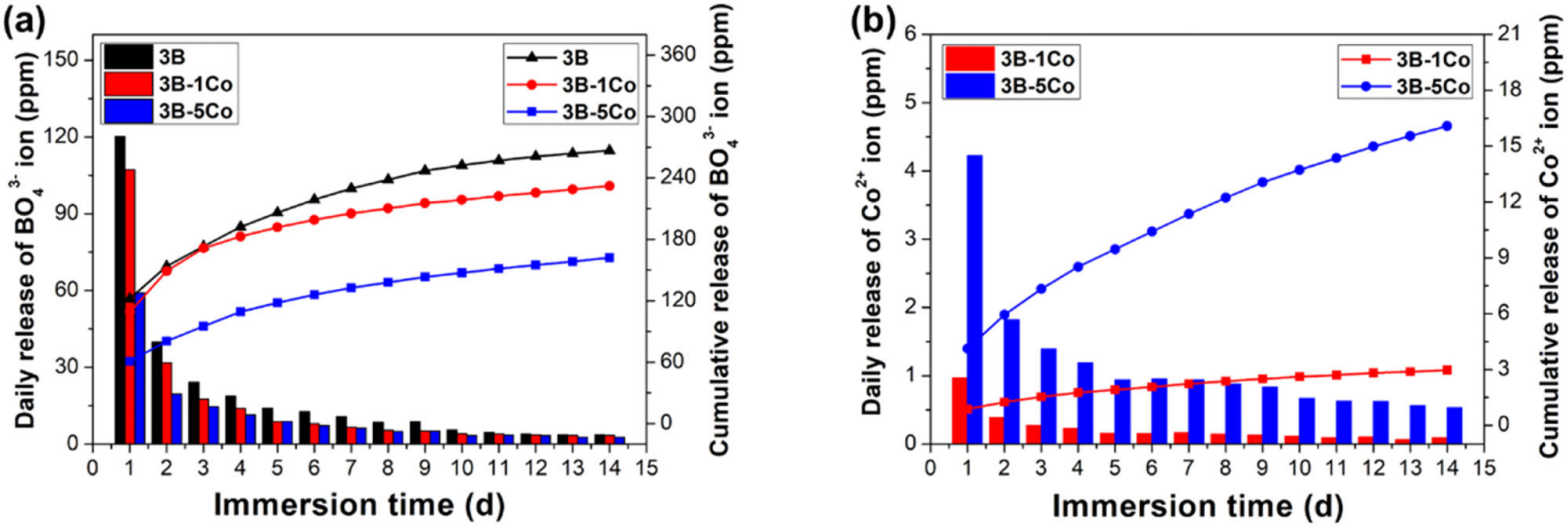
Concentrations of (**a**) B and (**b**) Co in SBF measured during immersion of different compositions of borate bioactive glass fibers for a period up to 14 days

The degradation of glass fibers in SBF results in the release of BO_4_^3−^ and Co^2+^ ions. Figure 4 shows the concentrations of B and Co in SBF measured during the immersion of BG, BG-1Co and BG-5 Co fibers for a period up to 14 days. An initial burst release of BO_4_^3−^ ions on the first day of immersion leads to a rapid increase of B concentration, reaching a maximum concentration of 120, 107 and 59 ppm for BG, BG-1Co and BG-5Co groups, respectively. Thereafter, the B concentration drop dramatically. It is noted that Co incorporation significantly inhibits the burst release of BO_4_^3-^ ions, especially the amount of BO_4_^3−^ ions released from the BG-5Co fibers is only half of that from the BG fibers on day 1.

The release profiles of Co^2+^ ions show the same general trend, but the release amount increases with increasing Co content. A rapid release on the first day is observed, reaching a plateau at Co concentration of 4.2 ppm for BG-5Co group and 1.0 ppm for BG-1Co group, which is followed by a slower release thereafter. The release of Co^2+^ ions appears to be almost complete within 5 days for BG-1Co group, while for BG-5Co group, a continuous release of Co^2+^ ions is detected, reaching a total concentration of 16 ppm over a reaction time range of 14 days.

#### Hydroxyapatite formation property

**Fig. 5:**
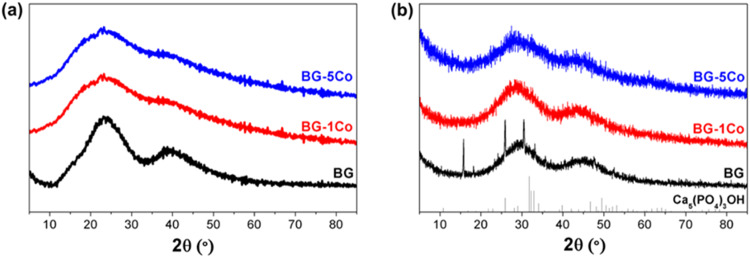
XRD patterns of different compositions of borate bioactive glass fibers after immersion in SBF at 37 °C for (**a**) 0 day and (**b**) 14 days

The conversion product of glass fibers in SBF was characterized by XRD and SEM-EDS. Figure 5a shows that all as-prepared BG, BG-1Co and BG-5Co fibers were amorphous. After 14 days of immersion, sharp diffraction peaks corresponding to a standard Ca_5_(PO_4_)_3_OH (JCPSD 09-0432) (HA) were detected for the BG group (Fig. 5b), suggesting the formation of HA crystals during the glass conversion process. Although the conversion products of BG-1Co and BG-5Co groups do not show obvious diffraction peaks, their XRD patterns reveal a shift in the amorphous halo compared to original glass, indicating the formation of an amorphous phase different from the original glass.

**Fig. 6:**
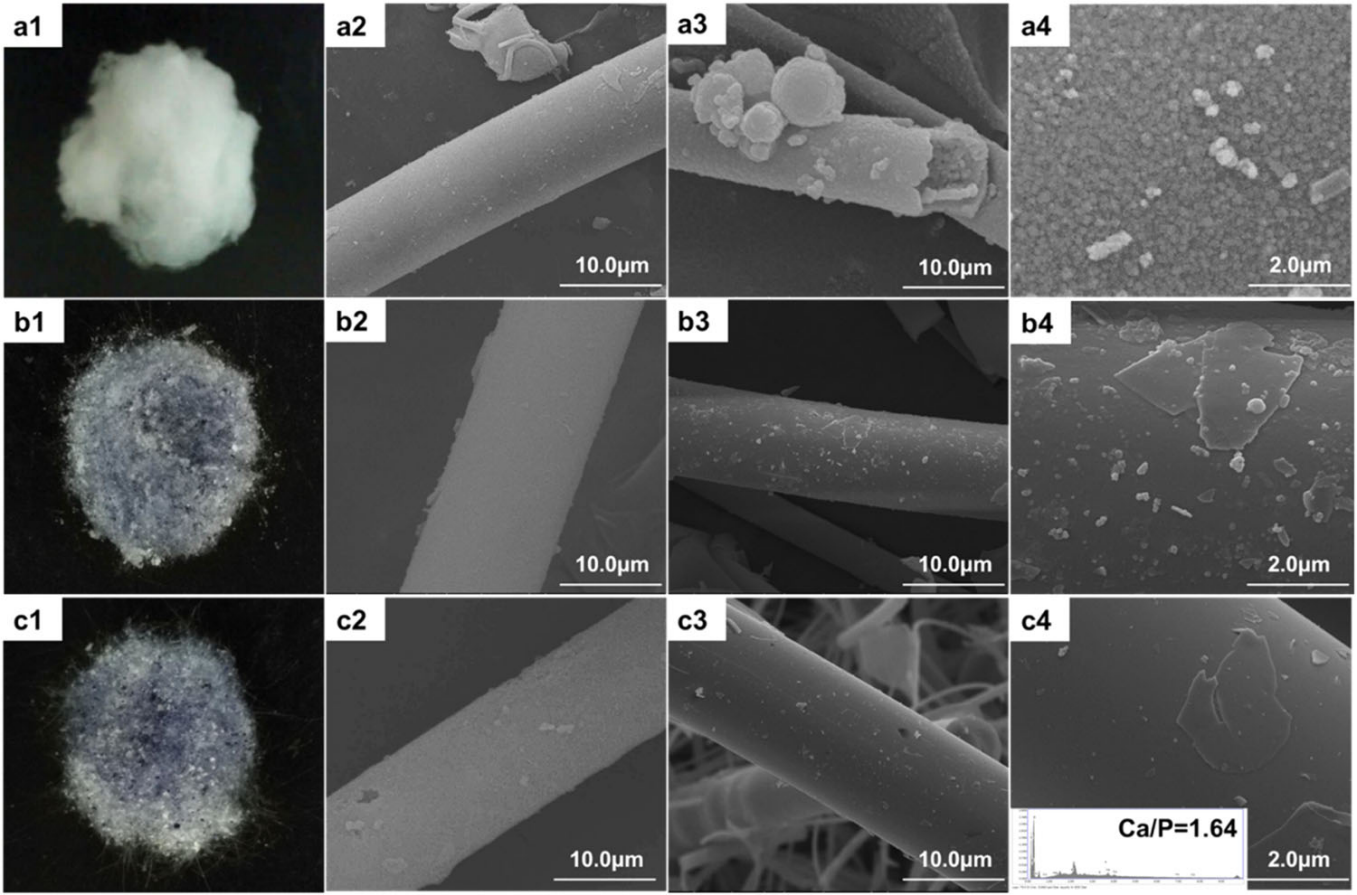
Photos of (**a1**) BG, (**b1**) BG-1Co and (**c1**) BG-5Co fibers; SEM images of (**a2**) BG, (**b2**) BG-1Co and (**c2**) BG-5Co fibers before immersion, SEM images of (**a3**, **a4**) BG, (**b3**, **b4**) BG-1Co and (**c3**, **c4**) BG-5Co fibers after immersion in SBF at 37 °C

SEM images of the glass fibers before and after immersion in SBF are shown in Fig. 6. As observed in Fig. 6a3, the initially solid BG fibers have become hollow, and no original glass is detectable. Higher magnification SEM image (Fig. 6a4) shows the hollow fiber wall is in fact built from spherical aggregates. Nevertheless, the Co-containing borate glass fibers still remain intact, and only a small amount of conversion products are detected (Fig. 6b3, b4). EDS analysis was performed to confirm the composition of conversion product, and the result revealed that the surface of the converted BG-5Co fibers was rich in calcium and phosphorus, with Ca/P atomic ratio of 1.64, which is approximately equal to the value of 1.67 for stoichiometric HA, indicating the formation of amorphous HA.

### In vitro cell experiments of the ionic dissolution products

#### Cytotoxicity and cell proliferation analysis

To ensure the safety of in vivo application and at the same time, considering the local accumulation of Co^2+^ ions under in vitro static culture condition, the borate glass fibers containing low levels of Co (BG-1Co, BG-2Co and BG-3Co) are used for further in vitro and in vivo investigations.

**Fig. 7:**
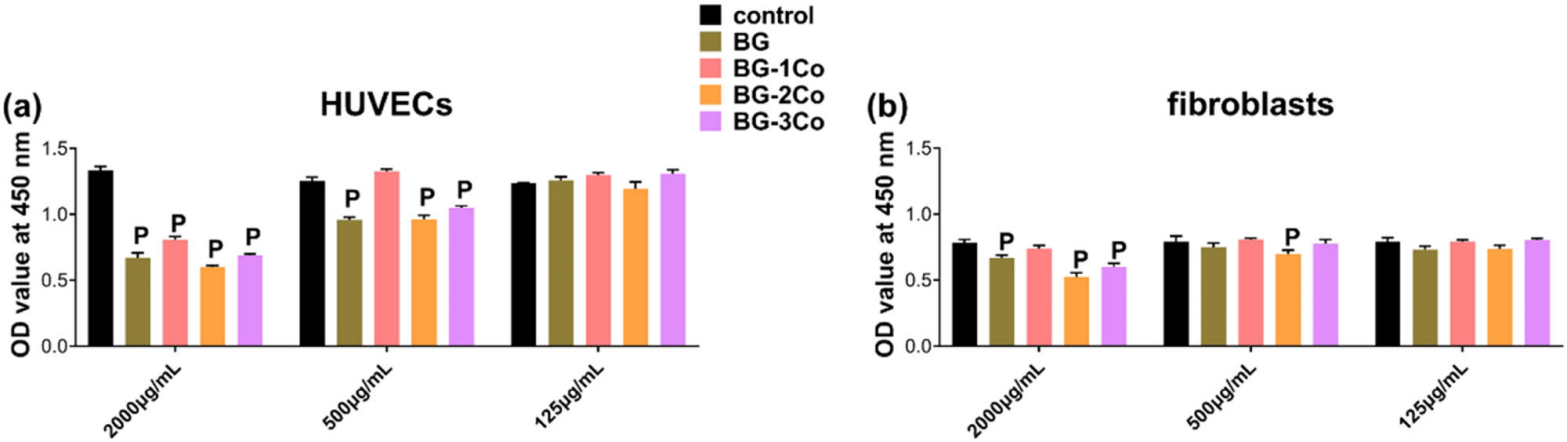
Quantitative analysis of the cell viability of (**a**) HUVECs and (**b**) fibroblasts after incubation for 3 days in increasing concentrations of ionic dissolution products from different compositions of borate bioactive glass fibers. (P: significant difference when compared to control in the same group (*P* < 0.05))

In vitro cytotoxicity of the BG and Co-containing borate glass fibers toward HUVECs and fibroblasts was measured using CCK-8 assay, and the results are shown in Fig. 7. Low concentration (125 μg/mL) of ionic dissolution products is not cytotoxic to both cells. When treating with increasing concentrations of ionic dissolution products, significant differences were observed between BG and Co-containing fibers. At 500 μg/mL, the absorbance OD values demonstrate a slightly increased cell viability in BG-1Co group, while other three experimental groups exert significant inhibitory effects on HUVECs compared to control group. Also, the cytotoxicity of BG fibers appears to be greater than that of the Co-containing fibers. At a higher concentration (2000 μg/mL), all four groups of extracts exhibit more evident decrease in cell viability. The fibroblasts appear to be more tolerant toward cytotoxic effects of the ionic dissolution products, and thus cell inhibition is only observed at higher concentration (2000 μg/mL). However, it is worth mentioning that, at this concentration, there is no statistically significant difference in OD values between BG-1Co and control groups. In addition, it is found that the highest toxicity is produced by BG-2Co group, not BG-3Co group.

**Fig. 8:**
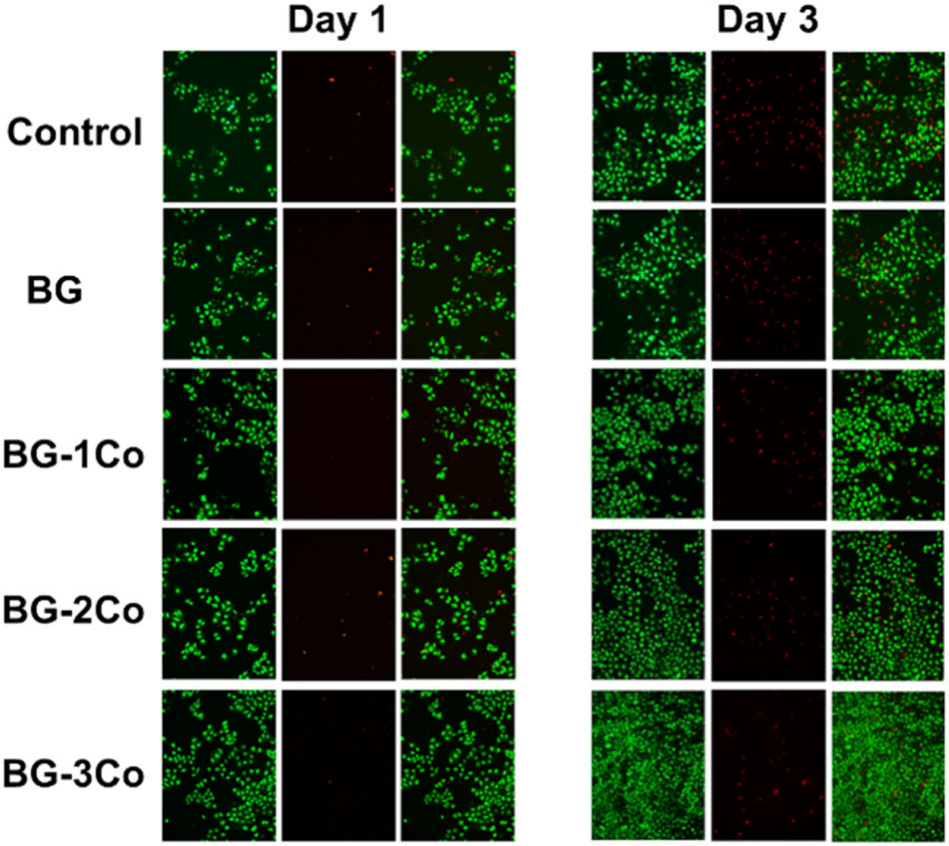
Confocal imaging of calcein-AM/PT stained HUVECs after incubation for 1 and 3 days with 125 μg/mL ionic dissolution products from different compositions of borate bioactive glass fibers. (Green is live cells, red is dead cells)

The in vitro cytotoxic effect of different extracts on HUVECs was further examined by live/dead cell double staining with calcein-AM/PI after 1 and 3 days of incubation. Figure 8 reveals that treating with 125 μg/mL extracts of Co-containing fibers results in increased cell proliferation and more live cells (green) with few dead cells (red) compared with BG and control groups. By comparing the number of live cells, it is found that the extracts of BG-3Co fibers exhibit stronger stimulatory effect on HUVECs proliferation.

#### Expression of angiogenesis-related protein

**Fig. 9:**
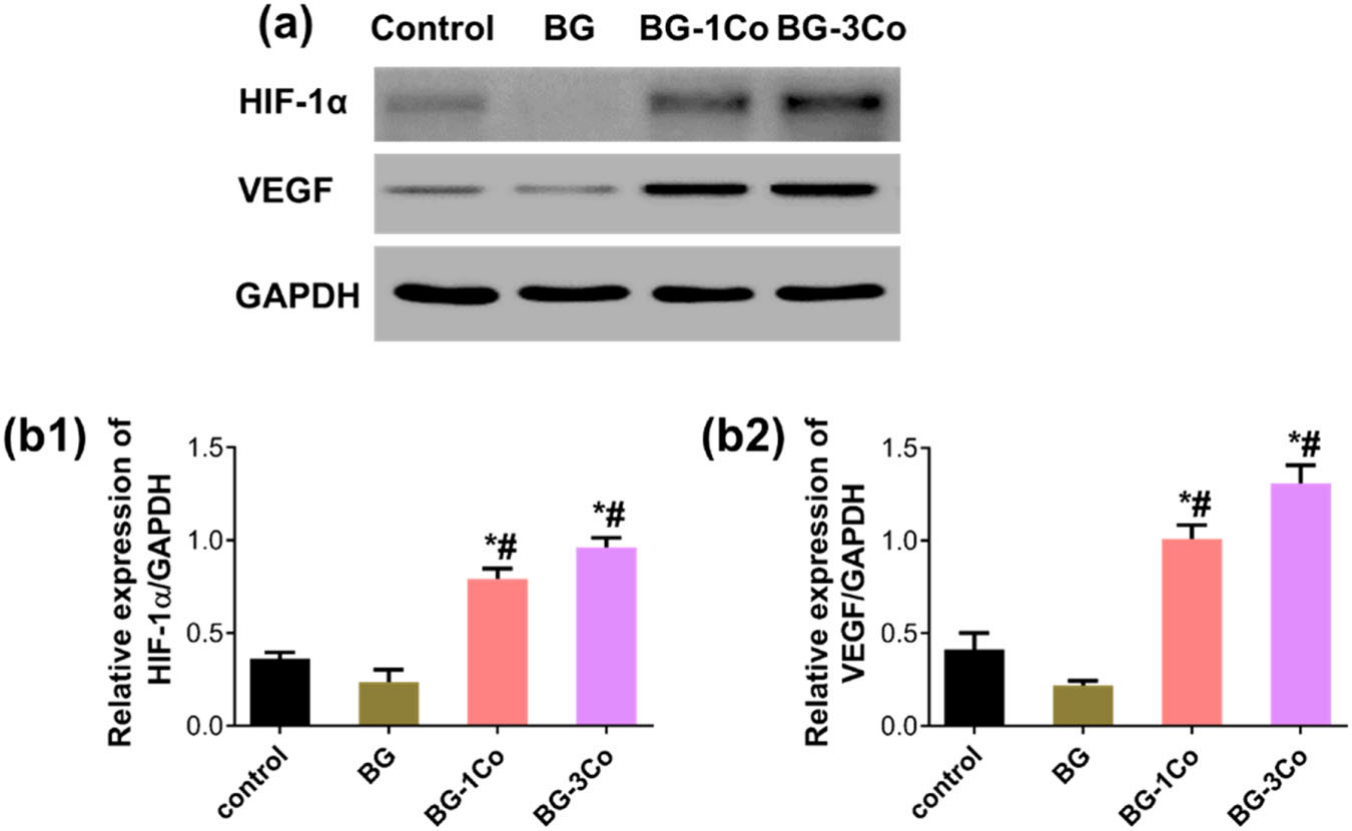
**a** Western blot analysis of HIF-1α and VEGF protein expression in the HUVECs incubated for 24 h in media containing 125 μg/mL ionic dissolution products from different compositions of borate bioactive glass fibers; quantification of (**b1**) HIF-1α and (**b2**) VEGF protein expression levels from the Western blot analysis. (*: significant difference when compared to control (*P* < 0.05), #: significant difference when compared to BG (*P* < 0.05))

The expression levels of HIF-1α and VEGF in HUVECs incubated for 24 h in media containing the ionic dissolution products of BG, BG-1Co and BG-3Co fibers were measured by western blot. As shown in Fig. 9, when incubated with 125 μg/mL extracts of BG fibers, the expressions of both HIF-1α and VEGF in HUVECs decrease compared to control group. Instead, significantly increased protein expressions were observed in the BG-1Co and BG-3Co groups. Especially the HIF-1α protein level induced by the BG-3Co fiber extracts is nearly 3-fold higher compared to control group, which furthermore promotes the secretion of VEGF protein in cells.

#### Cell migration

**Fig. 10:**
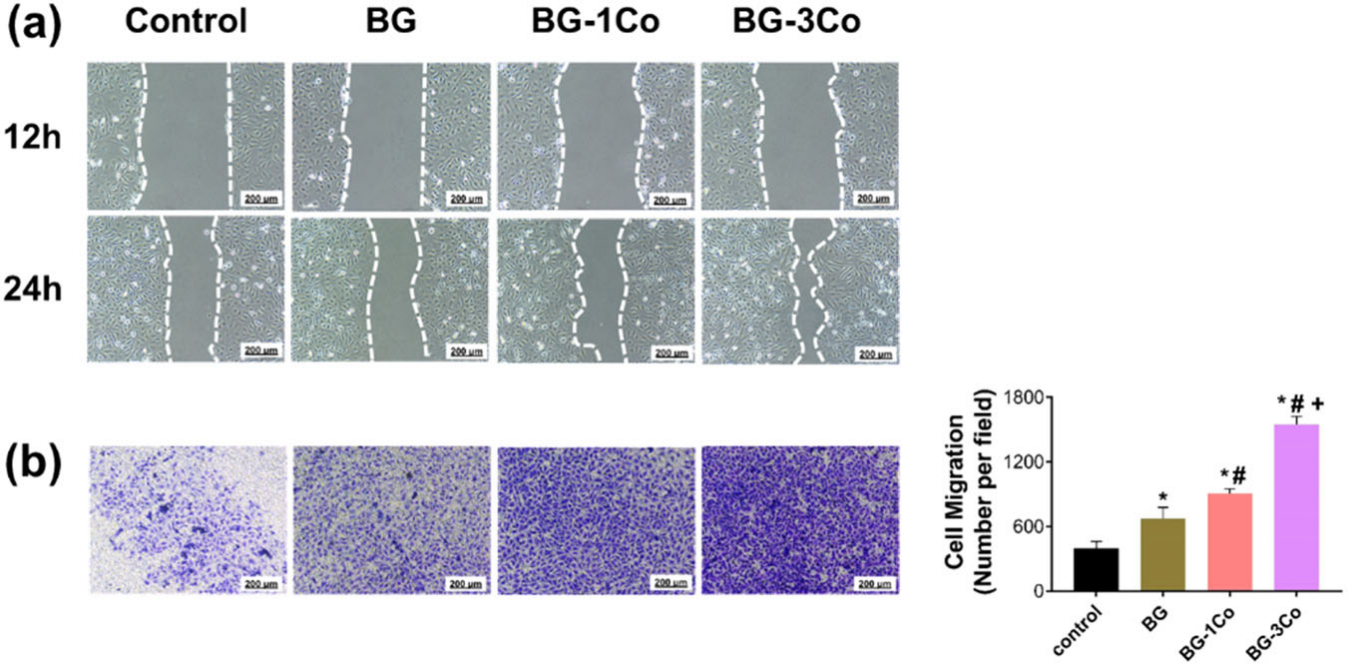
**a** Representative images of scratch-wound migration assay for HUVECs treated with 125 μg/mL ionic dissolution products from different compositions of borate bioactive glass fibers for 12 h and 24 h; **b** Images of Transwell assay and corresponding quantitative analysis for the HUVECs treated with 125 μg/mL ionic dissolution products from different compositions of borate bioactive glass fibers for 48 h. (*: significant difference when compared to control (*P* < 0.05), #: significant difference when compared to BG (*P* < 0.05), +: significant difference when compared to BG-1Co (*P* < 0.05))

The rapid migration of the HUVECs from the surrounding tissue to the center of wound can accelerate angiogenesis and promote wound healing. Therefore, the migration ability of HUVECs incubated with the ionic dissolution products of BG, BG-1Co and BG-3Co fibers were assessed by scratch-wound assay and Transwell assay. As shown in Fig. 10a, a significant difference is noticed in the cell migration distance between the Co-containing groups and control group. Comparatively, the positive impact of BG-3Co fiber extracts on cell migration is strongest among the other three experimental groups. Transwell assay and its quantitative results in Fig. 10b also show that the number of HUVECs migrated from the BG-3Co groups is highest, further suggesting the released Co^2+^ ions has a positive impact on accelerating cell migration.

#### Tube formation

**Fig. 11:**
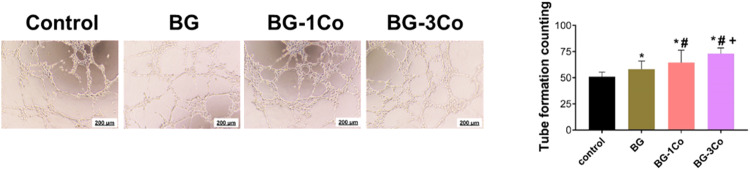
Images of tube formation assay and corresponding quantitative analysis for HUVECs treated with 125 μg/mL ionic dissolution products from different compositions of borate bioactive glass fibers for 6 h. (*: significant difference when compared to control (*P* < 0.05), #: significant difference when compared to BG (*P* < 0.05), +: significant difference when compared to BG-1Co (*P* < 0.05))

Furthermore, tube formation assay and corresponding quantitative analysis were performed to evaluate the angiogenic activity of the HUVECs incubated with different ionic extracts. As shown in Fig. 11, after incubation for 6 h, the BG-3Co group shows elongated and tube-like structures, while the HUVECs in control group form incomplete tubular networks. The tube number in Co-containing groups is significantly higher than that of the control group, and the tube number is proportional to the amount of CoO doping.

### In vivo animal experiments of Co doped borate glass fibers

#### Wound closure measurement

**Fig. 12:**
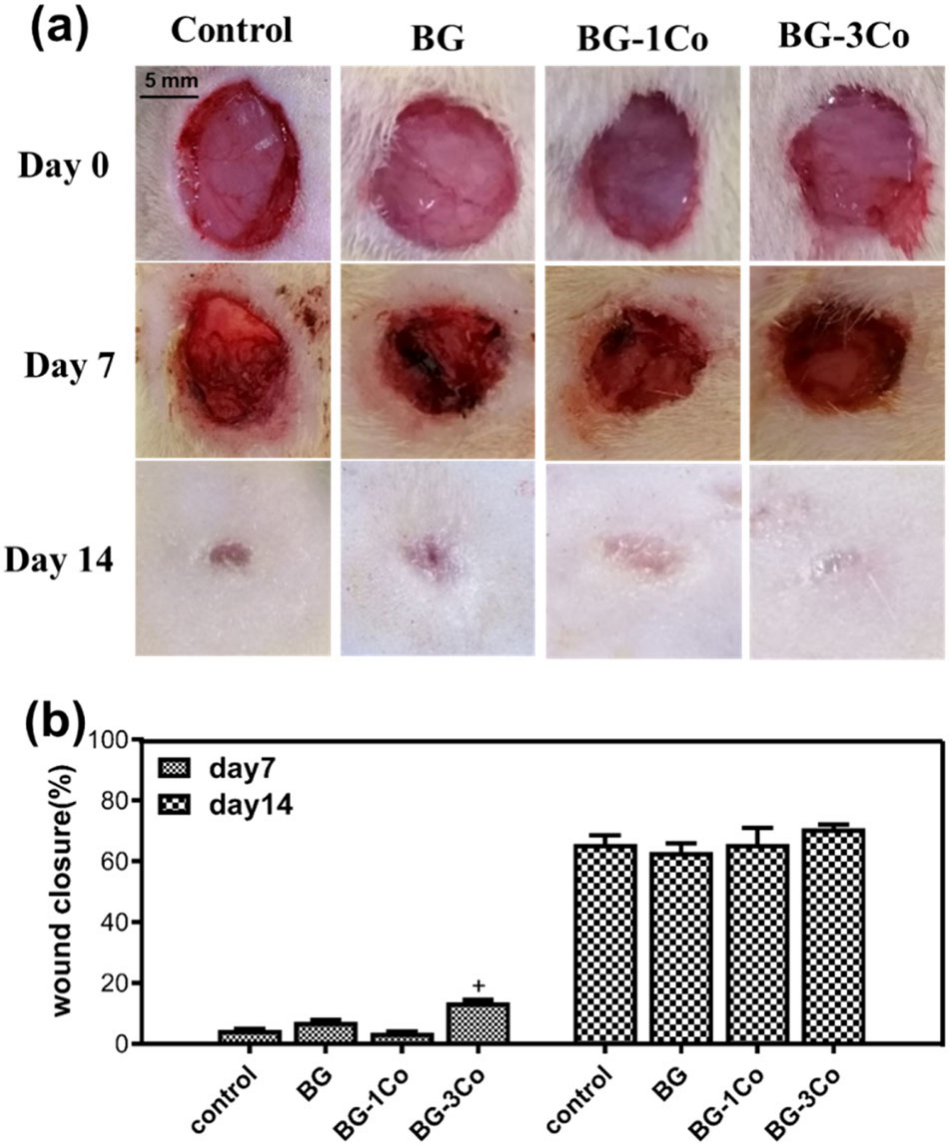
**a** Representative images of full-thickness skin defects in diabetic rats, the untreated defects (control) and the defects treated with different compositions of borate bioactive glass fibers on 0, 7 and 14 days post-surgery; **b** Quantitative analysis of wound closure. (+: significant difference when compared to BG-1Co (*P* < 0.05))

Wound healing potential of the Co-containing borate bioactive glass fibers was evaluated using STZ-induced diabetic rat model. Figure 12a shows representative images of the untreated defects and the defects treated with BG, BG-1Co and BG-3Co fibers on 0, 7 and 14 days post-surgery. While the wounds in all four groups become smaller with time, the wounds treated with Co-containing fibers close more completely than the control and BG groups. Quantified data (Fig. 12b) indicates that on day 7, the wound closure is significantly higher in the BG-2Co group compared to other groups, but on day 14 the highest wound closure is found in BG-3Co group with average closure rate of 70%.

#### Histological analysis

**Fig. 13:**
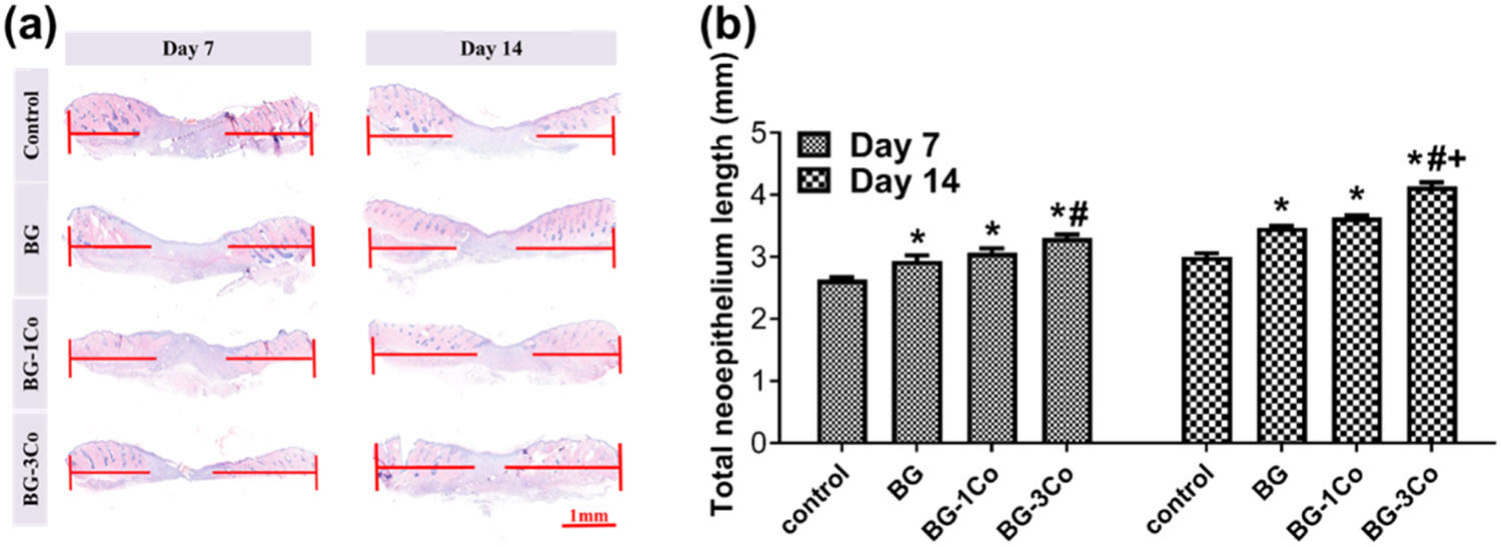
**a** Representative images of H&E staining of the untreated defects (control) and the defects treated with different compositions of borate bioactive glass fibers on 7 and 14 days, the new endothelium was marked by the red line, (Scale bar = 1 mm); **b** Total length of neo-epithelium width in the H&E stained sections. (*: significant difference when compared to control (*P* < 0.05), #: significant difference when compared to BG (*P* < 0.05), +: significant difference when compared to BG-1Co (*P* < 0.05))

To assess the healing quality of wound treated by Co-containing fibers, re-epithelialization were measured by H&E stains, and the results are shown in Fig. 13. On day 7, slight pro-epithelialization effect of BG-1Co and BG-3Co groups can be observed, as evidenced by the longer neo-epithelium width in the H&E stained sections (Fig. 13a). On day 14, all groups show further increased re-epithelialization. The total length of neo-epithelium measured based on H&E stains confirms that the wounds treated by BG-3Co fibers are almost completely covered by neo-epithelium. However, the re-epithelialization of wound treated by other groups is incomplete.

## Discussion

### Effect of Co incorporation on the network structure and properties of the borate glass

In FTIR spectra, the band at ~1220 cm^−1^ belonging to [BO_3_] units increases with increasing CoO content.

This change indicates that due to the higher ionic field strength of Co^2+^ than Ca^2+^, the interaction between Co^2+^ and (BO_3_)^−^ is higher than that between Ca^2+^ and (BO_3_)^−^. Hence, the high oxygen packing density of the glass network forms around the Co^2+^ [31], resulting in the strengthening of structure network of borate glass. In NMR spectra, the peak corresponding to [BO_3_] units was not found. It may because of the broadening of the NMR signals caused by the paramagnetism of Co^2+^. Another reason is that introduction of CoO into the borate glass by partially replacing CaO promotes the formation of [BO_4_] units at the expense of [BO_3_] units. The transformation contributes to strengthening the glass structure, preventing glass fibers from degrading too quickly. As [BO_4_] units are structural components, the release of BO_3_^3−^ ions can be used as a marker for glass degradation. After 10 days, almost no further increase of BO_3_^3−^ ions concentration in SBF, and hence it can be concluded that, with or without Co doping, the borate glass fibers nearly completely degraded within 10 days of reaction in SBF. The decreased release rate of BO_3_^3−^ from Co-containing fibers further confirms the role of Co^2+^ in strengthening glass structure network. It is indicated that Co-containing borate glass fibers have excellent degradation performance, which is of great importance for wound repair [19].

Controlling Co^2+^ ion concentration within the safe and effective range is the key to use. As for Co- containing fibers, the Co^2+^ ion released steadily within 5 ppm every day. It was reported that Co concentrations in the range of 10–14 ppm were within the biologically active limits. For example, treatment of human microvascular endothelial cells (HMEC-1) with 12 ppm CoCl_2_ resulted in binding of HIF-1, and hence transcriptional responses to hypoxia was mediated [32]. However, others also confirmed that around 10 ppm Co^2+^ ion release from Co-containing glass scaffolds had a toxic effect on endothelial cells [33]. Recent study showed that the Co concentration below 5 ppm is the safety window, and as low as 1 ppm CoCl_2_ exerted beneficial effects on angiogenesis and osteogenesis [34]. Our result shows that the borate glass fiber can provide a platform for Co^2+^ ion release, so that the release concentration of Co^2+^ ion can be controlled within the safe and effective range.

When immersed in SBF, the Ca^2+^ ion released from borate glass fibers and combined with PO_4_^3−^ group in the solution. After that, the Ca-P compound deposited on surface of glass fibers and gradually grows into Ca_5_(PO_4_)_3_OH crystals (HA). As shown in higher magnification SEM image of Co-free borate glass fiber (BG), the hollow fiber wall is built from spherical aggregates. Similar hollow structures have been found in the conversion products of highly reactive borate glasses with relatively low CaO content [35, 36]. Notably, compared with BG fibers, BG-1Co and BG-5Co fibers show more prominent inhibition effect on HA crystallization. This finding is consistent with previous reports [5], which confirmed that the addition of metal ions with smaller ionic radius relative to Ca^2+^ (such as Cu^2+^, Zn^2+^, Sr^2+^, Co^2+^, et al.) could substitute into the CaP layer in vitro or in vivo, thereby inhibiting the crystallization and growth of HA [5, 37–39]. For soft tissue repair, biomaterial with low HA formation capacity is required [19].

### In vitro biocompatibility and angiogenesis evaluation of the ionic dissolution products

It is well-known that the physiological and biochemical effect of elements is in a dose-dependent manner, and consequently, it is necessary to consider the amount of glass elements to achieve an optimum range of concentration below the toxic level. In vitro cytotoxicity assessed by CCK-8 assay shows that the BG-1Co glass fibers are biocompatible with osteoblast-like cell and HUVECs. Besides, it is noted that the decreased degradation rate of the Co-containing BG glass fibers reduced the toxicity induced by the released high concentration of borate ions, BO_3_^3-^, and rapid alkalization of the culture media.

The expression of HIF-1α and VEGF in HUVECs incubated with low concentration of BG extracts decreased when compared to control group. In the present study, the BG fibers are highly reactive due to their high B_2_O_3_ content and ultra-fine fiber diameter, and therefore, high rate of ion exchange reactions occur upon interaction of the fiber surface with cell culture medium, leading to burst ion release and significant increase in localized pH. Even though the ionic extracts at low concentration revealed no significant reduction of cell viability as evidenced in CCK-8 and live/dead cell staining experiments, the released ions and rapid pH increase might affect cell metabolism and function [40]. It has been found that an increase of pH can severely influence cell respiration provoking enzyme alteration and affect the diffusion of nutrients and gases to cell [41]. However, this effect is not usually observed in vivo because of a continuous dilution of the ions under dynamic conditions [42, 43].

On the contrary, when compared to control group, the expression of HIF-1α and VEGF in HUVECs incubated with low concentration extracts of Co-containing fibers increased. Co is well known to activate relevant HIF-1 pathways for regenerative processes by mimicking hypoxia condition. It was reported that Co can stabilize HIF-1α by binding to the iron center of HIF-hydroxylase [44, 45]. The stabilization promotes its accumulation in the nucleus of cells, where it is copolymerized with HIF-1β to form HIF-1, which in turn, activates the VEGF gene [46]. Within the non-toxic concentration range, more Co^2+^ ions were released from the BG-3Co fiber compared to the BG-1Co fiber at 24 h (Fig. 5b), which is likely to be the reason for the high expression of HIF-1α and VEGF proteins. Besides, more sustained BO_4_^3-^ ion release and pH alkalization also play a positive role in the upregulation of protein expression. VEGF is one of the most important pro-angiogenic mediators [47], and thus sufficient VEGF levels are believed to be essential for proper wound healing.

The migration ability and angiogenic activity of HUVECs incubated with the ionic dissolution products of BG-3Co fibers are highest, suggesting the released Co^2+^ ions has a positive impact on accelerating cell migration and promoting angiogenesis. Previous studies have confirmed that rapid functional angiogenesis is critical for the regeneration of damaged skin, because blood vessels are required for the delivery of immune cells and provide oxygen and nutrients for wound healing, and subsequently stimulate the consecutive events such as granulation tissue formation and collagen deposition [48, 49]. This process is mediated by pro-angiogenic modulators, primarily VEGF [50]. However, the impairment of HIF-1 pathway under high glucose conditions leads to significantly decreased expression of VEGF, which eventually results in inadequate healing of diabetic wounds. The present study demonstrates that the sustained release of Co^2+^ ions from the Co-containing fibers can promote the proliferation, migration and tube formation of the HUVECs by inducing a hypoxia response and upregulating the expression of angiogenesis-related proteins such as HIF-1α and VEGF.

### In vivo evaluation of diabetic wound healing

In vivo animal experiment results show that the wounds treated with Co-containing fibers close more completely than the control and BG groups, and the BG-3Co group is the best. Re-epithelialization describes the resurfacing of a wound with new epithelium. This process is one of the most essential part in wound healing process and completion of epithelialization has been used as a defining parameter of a successful wound closure [51]. Together, Co^2+^ ions released from the Co-containing fibers plays critical roles in promoting angiogenesis and re*-*epithelialization, two important parts of the proliferation stages in wound healing process. Besides, the anti-inflammatory and antibacterial activities of Co have also been extensively confirmed in vitro and in vivo [16, 52]. These functions all together effectively accelerate the diabetic wound repair process.

Borate bioactive glasses have demonstrated great potential in wound healing applications due to their: (1) high degradation rate, (2) the ability of B to stimulate angiogenesis, and (3) greater increase in local pH when compared with Si-based bioactive glasses, which is helpful for increasing antibacterial activity [53]. In this regard, wound dressing product (MIRRAGEN), composed of 1393B3 glass microfibers, has been approved by FDA for the treatment of first and second degree burns, traumatic wounds, and also diabetic, pressure, and venous ulcers [16]. Furthermore, the addition of biological active ions to a basic borate glass composition provides a viable approach to enhance the biological activity of the bioactive glasses. A typical example is the addition of Cu ions to 13–93B3 to induce an angiogenic effect. Cu-doped borate glass increased the rate of angiogenesis and accelerated the healing of full-thickness skin wound in rats compared with Cu-free base borate glass composition [54]. In the present study, we also confirmed that combining both Co and B ions was helpful for enhancing angiogenesis and re*-*epithelialization, and therefore accelerating the diabetic wound healing. Future research is needed to investigate the mechanism behind the synergistic effect between both ions.

## Conclusion

Co-containing borate bioactive glass fibers were successfully fabricated by partially replacing CaO in 1393B3 glass composition with CoO. The structural characterization showed that the substitution promoted the transformation of [BO_3_] into [BO_4_] units, thereby strengthening the structure network of borate glass, decreasing the dissolution rate and inhibiting the crystallization of surface CaP layers, which in turn, providing an ideal platform for sustained and controlled Co^2+^ ion release. In vitro results revealed that the fibers containing small amount of Co (1 mol% CoO) exhibited an improved cytocompatibility in comparison with Co-free borate glass fibers. Also, the Co^2+^ ion released from the Co-containing fibers can significantly enhanced the proliferation, migration and tube formation of the HUVECs by inducing a hypoxia response and upregulating the expression of angiogenesis-related proteins such as HIF-1α and VEGF. In vivo results demonstrated that the Co-containing fibers accelerated full-thickness skin wound healing in STZ-induced diabetic rat model by promoting angiogenesis and re-epithelialization. Collectively, the Co-containing borate bioactive glass fibers can be used as promising candidates for diabetic wound regeneration.
